# Dorsal Multi-Digit Heat-Press Injury: Staged Full-Thickness Skin Grafting with Range of Motion Tracking

**DOI:** 10.3390/reports9010069

**Published:** 2026-02-26

**Authors:** Shintaro Mitamura, Taisuke Sakamoto

**Affiliations:** 1 Department of Plastic and Reconstructive Surgery, Faculty of Medicine and Graduate School of Medicine, Hokkaido University, Sapporo 060-8638, Japan; 2 Department of Plastic and Reconstructive Surgery, Nikko Memorial Hospital, Muroran 051-0005, Japan

**Keywords:** case report, dorsal digits, full-thickness skin grafting, heat-press injury, range of motion

## Abstract

**Background and Clinical Significance:** Heat-press injuries of the hand can cause deceptively deep, progressive tissue damage, and dorsal multi-digit involvement carries a high risk of joint stiffness and scar contracture. **Case Presentation:** A 58-year-old left-hand-dominant woman sustained a dorsal heat-press injury affecting the left index to small fingers; we performed staged reconstruction with tangential debridement and artificial dermis placement (Day 9) followed by full-thickness skin grafting (FTSG) from the right infraclavicular region (Day 23), with supervised rehabilitation from Day 15 and active ROM resumed on postoperative day (POD) 6 after FTSG. **Conclusions:** At long-term follow-up (POD 821 after FTSG; ≈2.2 years), the reconstructed digits showed no hypertrophic scarring and achieved full finger motion with full fist formation; serial joint-specific active ROM tracking may enhance interpretability and comparability of outcomes in this uncommon but functionally critical injury pattern.

## 1. Introduction and Clinical Significance

Heat-press injuries (thermal-crush injuries) result from the combined effects of high temperature and mechanical compression. Compared with simple contact burns, compression intensifies heat transfer into deeper tissues and may compromise microcirculation, making early depth estimation and decisions regarding timing and extent of debridement difficult [1,2]. Even with a small total body surface area (TBSA), dorsal multi-digit injury spanning joints can lead to tendon adhesion, stiffness, and scar contracture with disproportionate functional impact [1,2,3].

Because these injuries are relatively uncommon, the evidence base consists mainly of small series and case reports [1,2,3,4,5,6,7,8]. Reports range from localized digit injuries to severe thermal-crush injuries extending proximally [8], and palmar injuries requiring specialized reconstruction [4]. A retrospective study further highlighted that insufficient extremity perfusion is an independent risk factor for amputation, underscoring the importance of vigilant assessment and timely escalation of care in heat-press mechanisms [5]. However, many publications provide limited joint-level functional data, hindering comparison across reconstructive and rehabilitation strategies. Serial joint-specific range of motion (ROM) tracking across multiple digits is particularly rare.

We present a dorsal multi-digit heat-press injury managed with staged tangential debridement and an artificial dermis followed by full-thickness skin grafting (FTSG), combined with intrinsic-plus positioning and structured rehabilitation. The distinguishing feature is serial documentation of active ROM of the distal and proximal interphalangeal (DIP and PIP) joints across the index to small fingers, together with long-term follow-up, showing durable scar quality and unrestricted function.

## 2. Case Presentation

**Figure 1:**
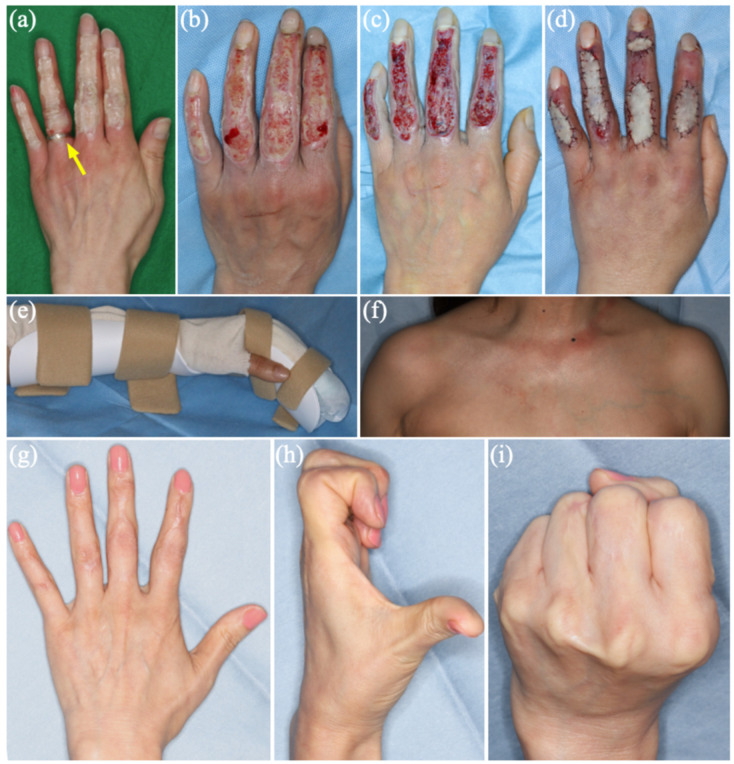
Clinical course of a dorsal multi-digit heat-press injury of the left hand. (**a**) Initial presentation (Day 0); a ring on the ring finger was removed promptly (arrow). (**b**) Before first-stage operation (Day 9). (**c**) Right after tangential debridement, showing deep dermal to full-thickness defects over the dorsum of the index to small fingers (Day 9). (**d**) Right after second-stage reconstruction with full-thickness skin grafting (Day 23). (**e**) Intrinsic-plus splint used for immobilization and positioning between dressing changes and rehabilitation sessions. (**f**–**i**) Long-term donor-site, scar, and functional outcomes at postoperative day (POD) 821 after full-thickness skin grafting, including the donor site (**f**), absence of hypertrophic scarring (**g**), full finger motion (**h**), and full fist formation (**i**).

A 58-year-old left-hand-dominant woman sustained an occupational heat-press injury when her left hand was trapped for several seconds in a dry-cleaning press machine heated to approximately 200 °C. The dorsal aspects of the index to small fingers were involved, and the estimated burn size was 0.5% TBSA. A ring on the ring finger was removed promptly (Figure 1a). Given the thermal-crush mechanism and concern for delayed demarcation, the patient was admitted for close monitoring and serial assessment. Radiographs showed no fractures. Hyperbaric oxygen therapy (HBOT) was initiated on Day 0 (nine sessions in total) as adjunctive care because thermal-crush injury may compromise microcirculation and tissue oxygenation; however, its clinical benefit could not be isolated in this specific case [9]. Immediate skin grafting was avoided because a stable, debrided wound bed is required, and the zone of injury can evolve after the thermal-crush injury. Because clinical depth can evolve after thermal-crush injury, a staged operative strategy was selected to achieve definitive closure while minimizing excision of potentially viable tissue [1,2,3]. HBOT was selected as an adjunct therapy based on clinical concern for microcirculatory compromise in deep thermal injury.

First-stage operation (Day 9): tangential debridement and artificial dermis. On Day 9, tangential debridement was performed to remove clearly devitalized tissue. Ideally, once nonviable tissue becomes evident, the first-stage procedure should be performed as early as possible (within the first week); however, limited access to general anesthesia due to staffing constraints at our regional hospital resulted in surgery on Day 9. The extensor tendons were not frankly exposed, and the paratenon was preserved, although portions of the dorsal defects approached the tendon level. After wound-bed preparation, a bilayer artificial dermis with a silicone layer (Terudermis^®^) was applied to stabilize the wound bed and facilitate subsequent definitive coverage (Figure 1b,c).

Second-stage operation (Day 23): full-thickness skin grafting. On Day 23, the silicone layer was removed and the wound bed demonstrated healthy granulation suitable for definitive coverage. Reconstruction was completed with full-thickness skin grafting (FTSG) to provide durable coverage and reduce late contracture across joint-spanning dorsal defects [2,4]. A split-thickness skin graft was considered; however, because the patient was a left-hand-dominant woman and the defects spanned multiple dorsal joints, we prioritized reduced secondary contraction and an aesthetically favorable match and therefore preferred FTSG when an adequate donor site was available. Full-thickness donor skin was harvested from the right infraclavicular region, carefully defatted, and the donor site was closed primarily. The infraclavicular region was selected to obtain a sufficiently large, pliable graft suitable for an exposed area; the medial upper arm was judged size-limited, and the groin was avoided due to potential pigmentation and color mismatch. The grafts were tailored to the dorsal digital defects and secured with standard dressing and protective positioning (Figure 1d).

Postoperative course: Supervised hand therapy was initiated on Day 15. Active ROM training was started; however, in the early phase, it was performed mainly as passive ROM because of pain (twice daily, every day), and an intrinsic-plus splint was worn between rehabilitation sessions. After FTSG on Day 23, rehabilitation was withheld for 5 days to prioritize graft take while splinting was continued. Therapy was resumed on postoperative day (POD) 6 after FTSG. Active ROM of each finger joint was measured using a goniometer with the patient seated and the shoulder, elbow, and wrist in a neutral position; measurements were performed 1–2 times per week by the same hand therapist at each time point. The splint was discontinued at discharge (POD 31 after FTSG), and outpatient hand therapy was continued once weekly thereafter. Sutures were removed between POD 8 and 11. Oral tranilast was administered for nine months as part of institutional scar management to mitigate early hypertrophic scarring (primarily in non-grafted areas) and was not considered a determinant of graft take or functional outcome. Several months after injury, hypertrophic scarring developed in part of the non-grafted proximal phalangeal region and was treated conservatively with topical steroid tape, with good response. The overall clinical timeline is summarized in Table 1. Serial active ROM of DIP and PIP joints of the index to small fingers was recorded at predefined time points and is summarized in Table 2, together with reference ROM values based on standardized measurement guidance [10].

Outcome: At the final follow-up (POD 821 after FTSG), the reconstructed dorsal fingers showed no hypertrophic scarring (Figure 1g). The right infraclavicular donor site demonstrated satisfactory long-term appearance (Figure 1f). Functional photographs confirmed full finger motion and full fist formation (Figure 1h,i). The patient reported no limitation in activities of daily living. At that time, she reported no pain or pruritus and was very satisfied with the outcome. The Vancouver Scar Scale (VSS; total 0–13, lower is better) score was 2 (vascularity 0, pigmentation 0, pliability 1, height 1), and the QuickDASH score was 0.

## 3. Discussion

Heat-press injuries differ from simple contact burns because combined thermal and compressive forces can produce deeper structural injury and delayed progression [1,2,3,4]. This uncertainty complicates early decisions regarding debridement and definitive coverage. In dorsal multi-digit injuries spanning DIP and PIP joints, a central goal is not only wound closure but also prevention of stiffness and contracture that may persist even after epithelialization. Accordingly, management should prioritize: (i) timely establishment of a stable wound bed, (ii) durable coverage that supports early mobilization, and (iii) structured rehabilitation with appropriate positioning.

Excessive early excision risks unnecessary sacrifice of viable tissue and may expose critical structures, whereas insufficient debridement risks persistent necrosis, infection, and delayed closure. A staged strategy can mitigate these competing risks by stabilizing the wound bed while allowing the zone of injury to declare itself [2,3]. In the present case, tangential debridement followed by an artificial dermis provided interim wound-bed stabilization, and definitive coverage with FTSG was performed once the bed was suitable, enabling structured rehabilitation without prolonged uncertainty regarding tissue viability. In a 20-year series analyzed using QuickDASH, Thuau et al. suggested that the first surgical excision is optimally performed approximately one week after injury, followed by serial excisions and definitive reconstruction, supporting a staged approach in selected cases [7].

Many reports provide qualitative statements (e.g., “good ROM”) or describe function at a single endpoint [2,4]. Such reporting limits comparison between reconstructive strategies and rehabilitation timelines, especially in multi-digit injuries where stiffness can differ by digit and joint. The principal contribution of this report is serial documentation of active DIP and PIP ROM across the index to small fingers (Table 2), anchored to standardized reference ROM guidance [10]. This joint-level trajectory is clinically interpretable for counseling (expected early limitation and recovery timeframe) and for benchmarking future reports.

Yapici et al. described a mechanism- and distribution-similar injury (multi-digit dorsal involvement of the proper digits (index to small fingers)) treated with repeated debridement during dressings and skin grafting in the third week, followed by physiotherapy [2]. Our case follows a comparable staged principle and similar timing to grafting. The key differentiators are (i) serial joint-level ROM across multiple digits and prolonged follow-up, and (ii) long-term confirmation (>2 years) of full finger motion and full fist formation with favorable scar quality. This improves interpretability beyond descriptive endpoints.

Additionally, Hihara et al. reported a similar case of a dorsal heat-press injury that was treated with immediate excision and coverage using a full-thickness skin graft (PSVN-FTSG) harvested from the groin within 20 h of the injury occurring [6]. At the 2-year follow-up, pigmentation of the graft and a slight extension deficit of the small finger DIP joint were noted [6]. Our staged approach—temporary coverage with an artificial dermis followed by conventional FTSG—resulted in favorable scar appearance and full range of motion, including full fist formation, at >2 years. Mechanistically, the artificial dermis may promote neodermis formation and provide a pliable wound bed that helps reduce extensor tendon adhesion and preserve tendon gliding during rehabilitation. Importantly, our serial joint-specific DIP/PIP ROM tracking provides a more granular functional trajectory than most prior reports, which may facilitate comparison across reconstructive strategies and rehabilitation timelines. Although graft selection for dorsal digital coverage remains debated, it essentially balances donor-site trade-offs (pigmentary change after STSG vs. a linear scar after FTSG) against secondary contracture risk at joint-spanning sites. In our case, FTSG provided an excellent aesthetic result without functional compromise. HBOT was used as an adjunct therapy; however, its independent contribution to the outcome cannot be determined in this single case, and objective microcirculatory assessment was not performed.

From a practical standpoint, management of dorsal multi-digit heat-press injury should be guided by two priorities: preservation of viable functional structures and early restoration of motion. When frank tendon or bone exposure is absent and the paratenon is preserved, a staged approach—limited tangential debridement with temporary wound-bed stabilization using an artificial dermis, followed by timely definitive coverage with skin grafting—can provide a stable, supple dorsal envelope that supports rehabilitation. Intrinsic-plus positioning maintained except during rehabilitation sessions, early supervised ROM restart after initial postoperative stabilization, and proactive scar control may further reduce the risk of stiffness and contracture. Importantly, serial joint-specific active ROM recording (DIP/PIP across digits), as demonstrated in this case, offers a simple and reproducible method to monitor recovery kinetics and to benchmark functional outcomes across similar cases.

Limitations: This is a single-case report, and outcomes reflect combined effects of staged reconstruction, rehabilitation, and scar management. ROM measurements were obtained clinically rather than via instrumented motion analysis. Nevertheless, serial joint-specific ROM documentation with long-term functional and scar outcomes provides practical information that is often missing in the heat-press injury literature.

## 4. Conclusions

Staged tangential debridement with an artificial dermis followed by FTSG, combined with intrinsic-plus positioning and structured rehabilitation, achieved durable functional recovery in a dorsal multi-digit heat-press injury. Serial joint-specific active ROM tracking may improve the quality and comparability of functional reporting in future case reports and small series.

## Figures and Tables

**Table 1:** Timeline of the clinical course.

Time Point	Event
Day 0	Admission; HBOT initiated (total 9 sessions); active finger motion encouraged.
Day 9	First-stage operation: tangential debridement + artificial dermis placement.
Day 15	Supervised hand therapy started; active ROM initiated (initially mainly passive due to pain; twice daily). Intrinsic-plus splint between sessions.
Day 23	Second-stage operation: FTSG.
POD 6 after FTSG	Rehabilitation resumed after temporary cessation to prioritize graft take.
POD 31 after FTSG	Discharge; splinting discontinued.
POD 821 after FTSG	Final follow-up; functional and aesthetic assessment.

FTSG, full-thickness skin grafting; HBOT, hyperbaric oxygen therapy; POD, postoperative day; ROM, range of motion.

**Table 2:** Serial active ROM of DIP and PIP joints (left index to small fingers).

Finger	Joint	Day 7	Day 15	Day 22	POD 6	POD 31	Reference ROM *
Index	DIP	50°/+10°	60°/+10°	60°/0°	60°/−10°	80°/+10°	80°/0°
	PIP	80°/0°	80°/0°	80°/0°	50°/−5°	100°/0°	100°/0°
Long	DIP	55°/+10°	60°/+10°	70°/0°	60°/0°	80°/+10°	80°/0°
	PIP	60°/0°	80°/0°	85°/0°	60°/−10°	90°/0°	100°/0°
Ring	DIP	55°/+5°	60°/0°	60°/0°	60°/0°	80°/+5°	80°/0°
	PIP	60°/0°	80°/0°	90°/0°	60°/−10°	95°/0°	100°/0°
Small	DIP	50°/0°	50°/0°	50°/0°	65°/0°	90°/0°	80°/0°
	PIP	70°/0°	80°/0°	75°/0°	50°/−10°	90°/0°	100°/0°

DIP, distal interphalangeal; PIP, proximal interphalangeal; POD, postoperative day; ROM, range of motion. Values are shown as maximum active flexion/active extension (degrees). Positive extension values indicate hyperextension beyond neutral, and negative values indicate an extension lag. * Reference ROM values are based on standardized measurement guidance [10].

## Data Availability

The original contributions presented in this study are included in the article. Further inquiries can be directed to the corresponding author(s).
